# Dynamics of ampicillin-resistant *Enterococcus faecium *clones colonizing hospitalized patients: data from a prospective observational study

**DOI:** 10.1186/1471-2334-12-68

**Published:** 2012-03-22

**Authors:** Maja Weisser, Evelien A Oostdijk, Rob JL Willems, Marc JM Bonten, Reno Frei, Luigia Elzi, Jörg Halter, Andreas F Widmer, Janetta Top

**Affiliations:** 1 Division of Infectious Diseases & Hospital Epidemiology, University Hospital Basel, Basel, Switzerland; 2 Department of Medical Microbiology, University Medical Center Utrecht, Utrecht, the Netherlands; 3 Julius Center for Health Sciences and Primary Care, University Medical Center Utrecht, Utrecht, the Netherlands; 4 Division of Clinical Microbiology, University Hospital Basel, Basel, Switzerland; 5 Division of Hematology, University Hospital Basel, Basel, Switzerland; 6 Department of Intensive Care Medicine, University Medical Center Utrecht, Utrecht, the Netherlands; 7 Division of Infectious Diseases & Hospital Epidemiology, University Hospital Basel, Petersgraben 4, CH- 4031 Basel, Switzerland

## Abstract

**Background:**

Little is known about the dynamics of colonizing *Enterococcus faecium *clones during hospitalization, invasive infection and after discharge.

**Methods:**

In a prospective observational study we compared intestinal *E. faecium *colonization in three patient cohorts: 1) Patients from the Hematology Unit at the University Hospital Basel (UHBS), Switzerland, were investigated by weekly rectal swabs (RS) during hospitalization (group 1a, n = 33) and monthly after discharge (group 1b, n = 21). 2) Patients from the Intensive Care Unit (ICU) at the University Medical Center Utrecht, the Netherlands (group 2, n = 25) were swabbed weekly. 3) Patients with invasive *E. faecium *infection at UHBS were swabbed at the time of infection (group 3, n = 22). From each RS five colonies with typical *E*. *faecium *morphology were picked. Species identification was confirmed by PCR and ampicillin-resistant *E. faecium *(ARE) isolates were typed using Multiple Locus Variable Number Tandem Repeat Analysis (MLVA). The Simpson's Index of Diversity (SID) was calculated.

**Results:**

Out of 558 ARE isolates from 354 RS, MT159 was the most prevalent clone (54%, 100%, 52% and 83% of ARE in groups 1a, 1b, 2 and 3, respectively). Among hematological inpatients 13 (40%) had ARE. During hospitalization, the SID of MLVA-typed ARE decreased from 0.745 [95%CI 0.657-0.833] in week 1 to 0.513 [95%CI 0.388-0.637] in week 3. After discharge the only detected ARE was MT159 in 3 patients. In the ICU (group 2) almost all patients (84%) were colonized with ARE. The SID increased significantly from 0.373 [95%CI 0.175-0.572] at week 1 to a maximum of 0.808 [95%CI 0.768-0.849] at week 3 due to acquisition of multiple ARE clones. All 16 patients with invasive ARE were colonized with the same MLVA clone (*p *< 0.001).

**Conclusions:**

In hospitalized high-risk patients MT159 is the most frequent colonizer and cause of invasive *E. faecium *infections. During hospitalization, ASE are quickly replaced by ARE. Diversity of ARE increases on units with possible cross-transmission such as ICUs. After hospitalization ARE are lost with the exception of MT159. In invasive infections, the invasive clone is the predominant gut colonizer.

## Background

Over the last decades *Enterococcus faecium *has emerged as an important nosocomial pathogen [1-3]. Molecular epidemiological studies using Multilocus Sequence Typing (MLST) [4] identified a genetic subpopulation of *E. faecium *clones that causes the majority of nosocomial infections and hospital outbreaks. It is characterized by resistance to various antibiotics, such as ampicillin (ARE), quinolones and vancomycin (VRE) [5] and acquisition of putative virulence genes [3,6-8]. This subpopulation is distinct from endogenous, genetically diverse and mostly ampicillin-susceptible *E. faecium *(ASE) colonizing the gastrointestinal tract of healthy individuals [9-12].

Prerequisite for infection is intestinal colonization [13]. Whether hospital-associated ARE originate from the commensal flora and outgrow endogenous *E. faecium *clones under antibiotic selection pressure or whether ARE are acquired in the hospital by transmission from a colonized environment (or other patients) is not clear [14], although the latter possibility has been strongly suggested [15,16].

In a prospective observational study we analyzed the within-patient dynamics and diversity of ARE clones colonizing high-risk patients on consecutive occasions during hospitalization and after discharge. Furthermore, from patients with an invasive ARE infection, genetic relatedness between the invasive and colonizing ARE was determined.

## Methods

### Study population

Three patients groups from different epidemiological settings were studied prospectively:

Group 1: All patients ≥ 18 years hospitalized between September 1^st ^and November 30^th ^2009 on a 13-bed hematology ward (for myeloablative chemotherapy or hematopoietic stem cell transplantation (HSCT)) of the University Hospital Basel (UHBS), a 600-bed tertiary care center in Switzerland were included (group 1a). Rectal swabs (RS) were obtained once weekly. Patients were treated in single rooms, supplied with laminar airflow, positive pressure and protective care. No antibiotic prophylaxis was administered besides trimethoprim/sulfamethoxazole for *Pneumocystis jirovecii*. In the 6 months after discharge, RS were obtained monthly during outpatient consultations (group 1b).

Group 2: All patients ≥ 18 years hospitalized between October 20^th ^and December 31^st ^2010 on a 30-bed Intensive Care Unit (ICU) of the University Medical Center Utrecht (UMCU), a 1042-bed tertiary care hospital in the Netherlands, had weekly RS. All patients received selective oropharyngeal decontamination (SOD) throughout ICU stay consisting of a mouth paste with non-absorbable anti-infectives (colistine, tobramycin and amphotericin B) [17]. Patients in groups 1 and 2 were eligible for analysis if at least three consecutive swabs were available.

Group 3: All patients ≥ 18 years with documented invasive infection with *E. faecium *hospitalized on any ward of the University Hospital Basel were included from September 1^st ^2009 until May 31^st ^2010. A single RS was obtained as soon as an invasive *E. faecium *infection was microbiologically confirmed.

### Microbiologic analysis

RS were obtained using a sterile cotton swab moistened with sterile 0.9% NaCl and immediately sent to the microbiological laboratory in a transport medium. RS were inoculated for 48 hours in Enterococcal Enrichment Broth (Becton-Dickinson, Cockeysville, MD, USA) supplemented with 75 mg/L aztreonam for suppression of Gram-negative bacteria. If positive (black coloration of the broth), 100 μl of well-mixed suspension were plated on Slanetz-Bartley Agar (Oxoid Ltd Basingstoke Hampshire, England) for 48 hours. Five colonies with a morphology typical for *E. faecium *were picked at random. If different morphotypes were present, five colonies from each morphotype were picked and grown on blood agar plates (BD trypticase soy agar with 5% sheep blood, Becton-Dickinson, Heidelberg, Germany). Bacterial lysates were prepared as described elsewhere [18]. An *E*. *faecium *species-specific PCR based on the *ddl *gene was performed to confirm species identity [19].

For invasive isolates, minimal inhibitory concentrations (MICs) for ampicillin and vancomycin were determined using a semiautomated microbroth dilution system (Micronaut, Merlin, Bornheim-Hersel, Germany). Isolates from RS were plated onto Enterococcosel Agar (Becton-Dickinson, Sparks, MD, USA) containing 60 mg/L aztreonam and 16 mg/L ampicillin to determine ampicillin susceptibility. Growth (indicating ampicillin resistance) was determined after 48 hours of incubation in aerobic conditions. Susceptibility was interpreted according breakpoints established by the Clinical and Laboratory Standards Institute (CLSI) for ampicillin (susceptible ≤ 8 mg/L, resistant ≥ 16 mg/L) and for vancomycin (susceptible ≤ 4 mg/L, intermediate 8-16 mg/L and resistant ≥ 32 mg/L) [20].

MLVA was performed on all ARE isolates from RS and invasive infections as described previously [21]. For isolates with incomplete MLVA-profiles, the Expand Long Template PCR system (Roche Diagnostics, Mannheim, Germany) was used on purified chromosomal DNA. MLVA types were assigned using the web-based database available at http://www.umcutrecht.nl/subsite/MLVA.

### Definitions

Duration of colonization by a clone was defined by the time between the first and the last detection. According to CDC-guidelines [22] two negative swabs were allowed in between. If three or more swabs were negative between two positive swabs, the second positive one was considered to represent a new acquisition. End of colonization was defined as the last positive swab, regardless of how many negative swabs were documented thereafter. If growth was documented only in a single swab, colonization time was defined arbitrarily as three days. Duration of antibiotic treatment was calculated in days with administration of an antibiotic regardless of class or changes in antibiotics.

### Statistical analysis

Longitudinal data was analyzed per hospitalization and per patient for outpatient-follow-up. In order to quantify the diversity of the different MLVA types of ARE, the Simpson's index of diversity (SID, Ridom EpiCompare^®^) [23,24] was used. The SID takes into account the number of different MLVA types in relation to the total number of isolates and was calculated for weeks 1-4 of hospitalization in groups 1a and 2. For group 1b SID was calculated for ARE isolates for intervals of 2-3 months. The SID was not calculated for later time points as the number of ARE isolates were too small (< 15) due to discharge of patients. For differences between hospitalization and ARE colonization rates, the Fisher's exact test was used. For comparison of ASE and ARE invasive infections the Fisher's exact test was used for categorical variables and the Mann Whitney test for continuous variables. A 2-sided *p*-value of < 0.05 was considered to be statistically significant. Analyses were performed using STATA™ software version 11 for Windows (StataCorp, College Station, TX, USA).

### Institutional review board (IRB)

All samples were part of the regular infection control surveillance program conducted by Hospital Hygiene and Infection Control. Approval from the local ethical committee (EKBB Rfe Number 208/09) was obtained.

## Results

### Patients and rectal swabs (RS)

Baseline characteristics of the 77 included patients are summarized in Table 1. Thirty-three of 34 patients admitted to the Hematology ward UHBS had at least 3 RS (group 1) during a total of 43 hospitalizations, of which 41 (95%) were admitted from home. Eight patients were already hospitalized at start of the study, and first swabs were, therefore, obtained after the first week of admission in these patients. Twenty-one of 27 patients from group 1a, who survived hospitalization, were included in outpatient follow-up (group 1b). The remaining 6 patients were lost to follow-up (of whom one was treated in another centre).

**Table 1:** Baseline Characteristics

	Hematology UHBS		ICU UMCU	INV UHBS
	**Group 1a**	**Group 1b**	**Group 2**	**Group 3**

**Patients**				
Screened patients; n	34	33	184	25
Included patients (≥ 3RS); n(%)	33 (97)	21 (64)	25 (14)	22 (88)^a^
Age, years; median (IQR)	55 (48-63)	53 (43-59)	55 (44-66)	69 (50-78)
Male sex; n (%)	18 (55)	11 (52)	19 (76)	13 (59)
Hospitalization during the previous year; n (%)				
- At the same institution	14 (42)	NA	10 (40)	11 (50)
- At another hospital	13 (39)	NA	NA	4 (18)
Death at end of follow-up, n (%)	6 (18)	12 (36)	10 (40)	7 (32)

**Episodes of hospitalization**	43	NA	29	22
Duration of hospitalization, days; median (IQR)				
- Hematology/ICU	27 (11-40)	NA	28 (18-48)	NA
- Any ward	33 (19-53)	NA	60 (42-78)	NA
Referral from another ward in the hospital, n (%)	2 (5)	NA	16 (55)	NA
Antibiotic treatment, n (%)	34 (79)	NA	28 (97)	NA
- Duration, days; median (IQR)	25 (15-38)		20 (14-33)	
- Antibiotics at admission, n (%)	14 (33)		6 (21)	
- Days to start since admission^b^,	6 (4-11)		2 (2-4)	
median (IQR)				

**Rectal swabs**; n	152	67	113	22
median per hospitalization (IQR)	3 (2-4)	3 (2-3)^c^	3 (3-5)	1
swabs containing *E. faecium*	59	24	62	21
*E. faecium *isolates; n	287	107	335	120
ARE; n (% of all isolates)	157 (55)	25 (23)	288 (86)	88 (73)
- MT159	85 (30)	25 (23)	151 (45)	73 (61)
- MT1	17 (6)	0	19 (6)	10 (8)
- MT 10	0	0	24 (7)	0
- MT12	10 (3)	0	47 (14)	0
- MT 282	19 (7)	0	0	5 (4)
- other ARE	26 (9)^d^	0	47 (14)^e^	0
MT159 in first swab, n (%)	3 (7)	3 (14)^f^	5 (17)	NA
MT159 acquisition during hospitalization, n (%)	3 (7)	1 (5)	9 (31)	NA

UHBS University Hospital Basel; UMCU University Medical Center Utrecht; ICU Intensive Care Unit; INV Invasive Infections, NA not applicable; RS rectal swab; ARE ampicillin-resistant *E. faecium*; ASE ampicillin-susceptible *E. faecium*; IQR Interquartile range;

^a ^patients with 1 RS at the time of invasive infection, 3 patients from group 1 are also included in 3; ^b ^in patients not treated with antibiotics at admission; ^c ^swabs performed during outpatient follow-up; ^d ^7 different MTs; ^e ^6 different MTs; ^f ^positive swab at time of discharge in outpatients

From a total of 184 patients admitted to the ICU UMCU (group 2), 25 were hospitalized 3 weeks or more and at least 3 RS could be obtained during 29 hospitalizations. In sixteen out of 29 ICU admissions (55%) the patient was directly transferred from another ward in the same hospital. From six patients the first RS was obtained later than the first week after ICU-admission.

At the UHBS 29 invasive infections with *E. faecium *were documented in 25 hospitalized patients (including 3 patients from group 1a) during the study period. In 22 patients a RS could be obtained at a median of 6 days after the first blood culture became positive (Interquartile range (IQR) 5-10).

Overall, 354 RS from 77 patients were analyzed. In about half of all RS (166 of 354, 47%) 849 *E. faecium *isolates were identified. ARE were present in 106 RS accounting for 558 isolates.

### Diversity of ARE

MLVA typing of the 558 ARE isolates yielded 16 different MLVA types (MTs) including 6 previously unidentified MTs. A remarkable predominance of MT159 was found (30%, 23%, 45% and 61% of all *E. faecium *isolates and 54%, 100%, 52% and 83% of all ARE in groups 1a, 1b, 2 and 3, respectively). Other predominant ARE clones are shown in Table 1. MT159 was present at the time of first sampling in 7% of hospitalizations in group1a and in 17% of hospitalizations in group 2. The acquisition rates of MT159 during hospitalization were 7% and 31% in groups 1a and 2, respectively. During outpatient follow-up MT159 was acquired in one patient.

### Dynamics of colonizing *E. faecium *clones

Table 2 shows the distribution of *E. faecium *in patients during follow-up: In group 1, in 22 of 33 (67%) patients *E. faecium *was isolated from a RS at least once, 13 (40%) patients had at least one ARE. In group 2, 23 of 25 (92%) patients had at least one RS positive for *E*. *faecium *and in 21 (84%) patients an ARE was detected at least once. The highest proportion of patients with ASE only was in group 1b (Table 2). A minority of patients had one ARE clone only during the follow-up (4, 2 and 12 in groups 1a, 1b and 2, respectively). In 9 patients from group 2 (36%) only MT159 was documented. In 6 out of 33 patients from group 1, a swab containing an ASE was followed by an ARE positive swab, which was interpreted as an ASE to ARE replacement. Replacement of ARE by ASE occurred in only 1 patient from group 1 and 2 patients from group 2.

**Table 2:** Within-patient distribution of *E. faecium *isolates

	Hematology UHBS		ICU UMCU
	**Group 1a**	**Group 1b**	**Group 2**

**Patients, n**	33	21	25
- with ARE, n (%)	13 (40%)	3 (14%)	21 (84%)
- with only ASE, n (%)	9 (27%)	9 (43%)	2 (8%)
- with no *E. faecium*, n (%)	11 (33%)	9 (43%)	2 (8%)
Patients with only one MT of ARE			
- MT159	1	2	9
- Other ARE	3	0	3
Patients with replacements^a ^of *E. faecium*			
- ASE to ARE	6	0	0
- ARE to ASE	1	0	2
- ARE change of MT	2	1	7

UHBS University Hospital Basel; ICU Intensive Care Unit; UMCU University Medical Center Utrecht; ARE ampicillin-resistant *E. faecium*; ASE ampicillin-susceptible *E. faecium *^a ^Replacement of an ampicillin-susceptible by an ampicillin-resistant clone in consecutive swab or vice versa or replacement of an ARE by a different ARE MT clone

**Figure 1:**
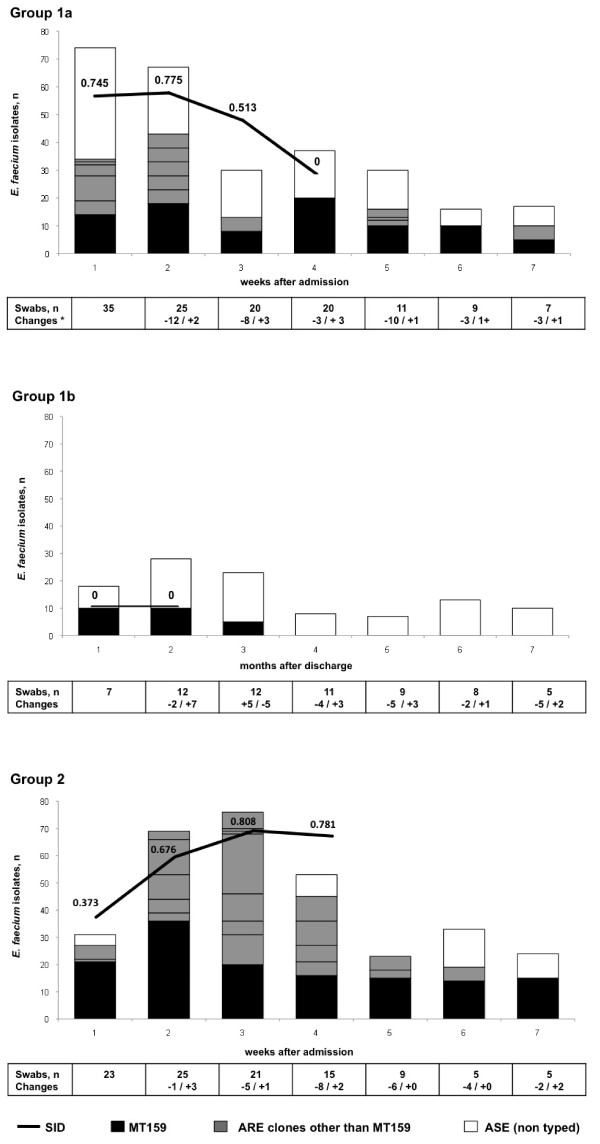
**Dynamics of colonizing *E. faecium *clones**. Bars indicate the proportion of *E*. *faecium *isolates among the collected isolates at different time points; black color indicates MT159 clones, dark grey ampicillin-resistant non-MT159 clones and white color indicates ampicillin- susceptible non-typed *E. faecium *isolates. The black line shows the Simpson's index of diversity for ARE during the first 4 weeks in hospitalized patients and during months 1 & 2, 3 & 4 and 5, 6 & 7 in hematological outpatients. Confidence intervals are indicated in the text. Numbers of swabs are shown below the graph. Fluctuation in numbers of swabs is due to missing admission swabs in 8 hospitalizations (in which patients have been on the ward before start of the study), to discharge of patients at different time points and to lack of 3 swabs during hospitalization.

Table 3 shows the dynamics of ARE clones per hospitalization and outpatient treatment: In group 1a, the number of *E. faecium *isolates cultured per week declined from 74 (34 ARE, 40 ASE) in week 1 to 37 (20 ARE, 17 ASE) in week 4. The SID of MLVA-typed ARE increased slightly but not significantly from 0.745 [95%CI 0.657-0.833] in week 1 to 0.775 [95%CI 0.684-0.866] in week 2 probably due to acquisition of ARE clones and then dropped significantly to 0.513 [95%CI 0.388-0.637] in week 3 and to zero in week 4 with loss of all ARE except MT159 in this last time point (Table 3 Figure 1). The decrease in SID was accompanied by a substantial loss of ASE during the first 2 weeks of hospitalization. Acquisition of ARE clones was documented during 8 (19%) of 43 hospitalizations, mostly (n = 5) occurring between week 1 and 2.

**Table 3:** Diversity of ARE clones over time

	Week 1	Week 2	Week 3	Week 4
**Group 1a: Hospitalizations of hematological inpatients UHBS (**n = 43)				
*E. faecium *isolates, n	74	67	30	37
ARE, n	34	43	13	20
ASE, n	40	24	17	17
SID of ARE	0.745	0.775	0.513	0
95% Confidence Interval	[0.657-0.833]	[0.684-0.866]	[0.388-0.637]	[0.0-0.0]

	**Month 1 & 2**	**Month 3 & 4**	**Month 5 & 6 & 7**	

**Group 1b: Hematological Outpatients UHBS **(n = 21)				
*E. faecium *isolates, n	46	31		30
ARE, n	20	5		0
ASE, n	26	26		30
SID of ARE	0	0		
95% Confidence Interval	[0.0-0.0]	[0.0-0.0]		

	**Week 1**	**Week 2**	**Week 3**	**Week 4**

**Group 2: Hospitalizations of Intensive Care Unit UMCU (**n = 29)				
*E. faecium *isolates, n	31	69	76	50
ARE, n	27	69	76	45
ASE, n	4	0	0	5
SID of ARE	0.373	0.676	0.808	0.781
95% Confidence Interval	[0.175-0.572]	[0.58-0.772]	[0.768-0.849]	[0.725-0.837]

UHBS University Hospital Basel; UMCU University Medical Center Utrecht; SID Simpson's Index of Diversity; ARE ampicillin-resistant *E*. *faecium*; ASE ampicillin-susceptible *E. faecium*

Colonization with MT159 was detected at the first swab occasion in three (7%) of 43 hospitalizations involving three different patients; two of these patients had suffered from an invasive infection with *E. faecium *MT159 during the previous year. In three hospitalizations MT159 was acquired during stay at the hematology; two of these patients had been transferred to and from an ICU. In all six hospitalizations MT159 colonization persisted until discharge, whereas 7 non-MT159 ARE clones disappeared before discharge in 5 patients.

Of the 21 patients of group 1b three patients remained colonized with ARE during the first three months after hospital discharge. All these ARE were MT159.

In the ICU (group 2), almost all *E. faecium *isolates were ARE. The SID of ARE isolates increased significantly from 0.373 [95%CI 0.175-0.572] at week 1 to a maximum of 0.808 [95%CI 0.768-0.849] at week 3. During week 4 SID decreased slightly to 0.781 [95%CI 0.725-0.837]. ARE were detected in the first swab in eight (28%) of 29 hospitalizations; five belonged to MT159; in six admissions the patient was transferred from another ward to ICU. There were 24 ARE acquisitions (comprising 8 different clones) during 29 ICU admissions (83%) including 9 acquisitions with MT159 (31%). Most clones were acquired during the first 3 weeks (Figure 1). In only 4 patients (7 hospitalizations) no ARE were detected. Disappearance of a clone at the time of discharge occurred for 14 (70%) of 20 non-MT159-ARE clones in 11 patients and for 4 (29%) of 14 MT159 in 14 patients (*p *< 0.02).

Antibiotic consumption was high in all hospitalized patients (Table 1): In group 1a, during 34 hospitalizations out of 43 (79%) broadspectrum antibiotics were administered for a median duration of 25 days (IQR 25-38), which corresponds almost to the whole duration of hospitalization. In group 2, administration of antibiotics was even higher (28 out of 29 hospitalizations (97%)) with a median duration of 20 days (IQR 14-33). During hospitalizations without administration of antibiotics, acquisition of ARE was low (1 out of 9 hospitalizations (11%) in group 1a and 0 out 1 hospitalization in group 2). In contrast, during hospitalizations with antibiotic treatment, acquisition of ARE was more frequent (13 out of 34 hospitalizations (38%) in group 1a and 22 out of 28 hospitalizations (79%) in group 2).

### Invasive Infections with *E. faecium*

The majority (91%) of 22 invasive isolates was obtained from a blood culture (Table 4). All 22 isolates were susceptible to vancomycin, 16 were resistant to ampicillin. Among invasive infections with ARE 13 of 16 (82%) were caused by MT159. Other isolates belonged to MT1 (n = 2; 12%), MT282 (n = 1, 6%). An identical MLVA clone in the RS was present in all 16 patients with invasive ARE infection. Two patients with ARE infection were co-colonized with ASE. Invasive ARE infections were mostly monomicrobial (69%), related to central venous catheter or vascular grafts (75%) and occurred after a median of 20 days after admission. Most infections with MT159 (69%) occurred during or after stay in ICU. ASE infections were more often polymicrobial (67%), resulted from cholangitis (83%) and occurred early (median of 3 hospitalization days). In these patients, no ARE were found in the RS.

**Table 4:** Invasive Infections with *E. faecium*

	ARE n = 16	ASE n = 6	p-value
**Source of microbiological sample**			
- blood, n (%)	14 (88)	6 (100)	0.519
- abscess-drainage, n (%)	2 (12)	0	
**Polymicrobial infection, n (%)**	5 (31)	4 (67)	0.155
**Underlying disease, n (%)**			
Hematological malignancy	5 (31)	1 (17)	
Hepatobiliary disease	2 (13)	5 (83)	
Intestinal disease (M. Crohn)	1 (6)	0	0.031
Vascular graft	3 (19)	0	
Other^a^	5 (31)	0	
**Clinical presentation, n (%)**			
CVC-related infection	5 (31)	0	
CVC colonization	4 (25)	1 (17)	
Infection of vascular prosthesis	3 (19)	0	0.046
Cholangitis	2 (13)	5 (83)	
Other^b^	2 (13)	0	
Days since admission, median (IQR)	20 (10-29)	3 (1-11)	0.089
Hospitalization on Intensive Care, n (%)	9 (56)	2 (33)	0.318
**Death, n (%)**	6 (38)	1 (17)	0.35

CVC central venous line; IQR interquartile range, ^a ^chronic obstructive pulmonary disease (2), intravenous drug use (1), lung cancer (1), spondylodiscitis (1); ^b ^intraabdominal abscess/peritonitis (1), unknown (1)

## Discussion

In this study we have demonstrated the predominance of a single clone (MT159) of *E*. *faecium *colonizing hospitalized patients and causing invasive infections in two hospitals in Switzerland and the Netherlands. In hematological patients admitted directly from home ASE carriage was rapidly replaced by ARE, mainly MT159. In these patients ARE carriage disappeared after hospital discharge with the exception of MT159, which persisted in three patients. In patients from ICU ARE were abundant already on admission and increased in diversity during ICU stay.

In the two epidemiological settings examined, dynamics of ARE were different. In hematological patients the SID at admission was high (0.745) compared to patients from ICU (0.373). In parallel the amount of ASE was much higher in hematological patients. This can partly be explained by differences in baseline characteristics: Hematology patients are predominantly admitted directly from home and thus have been transferred from another ward less frequently than patients from ICU (5% vs 55%, respectively, *p *< 0.01). As a result, ARE prevalence, which is typical for hospital environments, at the time of the first swab tended to be lower (19% versus 28% respectively, *p *< 0.05). Differences in the prevalence of ARE carriage in the community cannot be excluded, but seem less probable. In the Netherlands the proportion of ARE in 650 community-derived fecal samples was 3% in 2007 [21] and was unchanged in 2010 despite the increase of ARE in hospitals (J. Top, personal communication).

During hospitalization (week 3) the SID decreased significantly in haematological patients due to loss of ARE other than MT159 and a few acquisitions of MT159. In contrast, in ICU patients, the SID increased significantly during the first week of hospitalization due to acquisition of different ARE clones. Acquisition rates of ARE during hospitalization were markedly higher in ICU (83%) than in hematological patients (19%). This could be related to protective care measures in single rooms for hematology patients, while ICU patients were, at the time of the study, treated in 5-bed-rooms, increasing the chance of cross-transmission. In both study groups, most acquisitions occurred at week 2 and 3 of hospitalization. As we swabbed only once weekly the exact time point of acquisition might have been earlier. In both groups antibiotic consumption was high (group 1a 79%, group 2 97%). A proper correlation between acquisition of ARE and antibiotic consumption was unfortunately not feasible due to low numbers. Thus the increase in ARE could also correspond to a selection of pre-existing ARE outnumbering ASE. In this study all patients in ICU received SOD, an oral antibiotic-containing paste, which may promote colonization in ARE [25,26]. Additionally, ARE epidemiology at the UMCU could be characterized as endemic with clonal diversification and polyclonal expansion since 2000 [27], whereas an increase in invasive ARE infections in the University Hospital Basel was observed only after 2004 (data not shown).

Our findings of a decrease in diversity of *E. faecium *isolates at later time points during hospitalization in hematology patients, is in agreement with a recently published study: In 8 patients admitted from home to a neurosurgical ICU in Madrid, Spain, a newly generated index quantifying daily diversity showed a decrease of *E. faecium *diversity with persistence of nosocomial clones during hospitalization [14]. We used the SID to quantify diversity based on 5 at random picked *E. faecium *isolates from weekly rectal swabs. The SID is an established measure of biodiversity [24], but is usually used in larger sample sizes. One could argue that numbers of isolates - ranging from 17 to 76 per time point - were too small to adopt such an index. We decided therefore to calculate the SID only for the time points with at least 15 available isolates, which was the case for the first four weeks of hospitalization.

During follow-up, 55% of patients with *E. faecium *colonization carried more than 1 ARE clone. During 17 hospitalizations, 25 ARE clones were no longer detected at the last swab before discharge (7 in group 1a and 18 in group 2). This could be due to real acquisition and subsequent loss of clones, or to the low sensitivity of the RS culture method. The sensitivity of directly inoculating swabs on selective agar was estimated between 58-80% for VRE [28,29] with a detection limit of 4 log_10 _colony-forming units of VRE/g feces [29]. To enhance sensitivity, we used an enrichment step before plating [30]. The 'loss' of ARE after discharge might represent a reduced selection pressure with bacterial loads decreasing to levels below detection limits.

MT159 persisted in patients from both hospitals (100% in group 1a, 71% in group 2). After discharge (group 1b) MT159 was found in 3 patients and persisted throughout follow-up in 2 patients. Higher persistence of ARE relative to ASE clones has been reported [14].

Since 2004, MT159 isolates, represented by ST78, has emerged as an epidemic *E. faecium *clone causing invasive infections and hospital outbreaks in many hospitals worldwide [15,21,31,32]. The reason for its particular success in spread and infection is not understood. Although acquisition of multiple antibiotic resistance genes and genes that enhance colonization and infection capacities might contribute, such acquisitions appear not unique for MT159 [3,33]. Possibly, the high propensity to persist contributes to the ecological success of this clone.

MLVA has been proposed as a tool to study genetic relatedness in *E. faecium *with comparable discriminatory index compared to MLST [18], although other studies indicated that MLVA might be less discriminatory than PFGE and MLST [32,34]. In a study from 2008 we showed that PFGE and MLVA produced highly concordant results when assigning genotypes to nosocomial *E. faecium *isolates [35]. Because MLVA is faster than PFGE and MLST, which was an important criterion when analyzing such a high amount of isolates, we have selected MLVA for this study.

At the patient level, there was 100% concordance in *E. faecium *MLVA-types among ARE isolates causing invasive infections and isolates from rectal swabs. Most invasive ASE infections were polymicrobial (67%) and of hepatobiliary origin (83%) in patients admitted from home in contrast to ARE infections, which were predominantly monomicrobial (69%) and associated with a haematological malignancy (31%), ICU stay (56%) and presence of vascular foreign bodies (75%). This underscores, the concept that nosocomial *E. faecium *infections are predominantly caused by ARE acquired during hospitalization and that endogenous ASE only sporadically cause invasive infections.

## Conclusion

In hospitalized high-risk patients MT159 is the most frequent colonizer and cause of invasive *E. faecium *infections. During hospitalization, ASE are quickly replaced by ARE. Diversity of ARE increases on units with possible cross-transmission such as ICUs. After hospitalization ARE are no longer detectable with the exception of MT159 which was able to persist.

In patients with invasive infections, the invasive clone is also the predominant colonizer in the gut. Invasive ARE infections were mostly monomicrobial, related to vascular devices and appear mostly in patients with long hospitalizations

## Competing interests

The authors declare that they have no competing interests.

## Authors' contributions

MW conceived the study, participated in its design, carried out the microbiological analysis (cultures, resistance testing, MLVA typing) and drafted the manuscript. EAO participated in the study design, the microbiological sampling in Utrecht and analysis of the data. RJLW participated in the study design and coordination and helped to draft the manuscript. MJMB participated in the study design, analysis of the data and helped to draft the manuscript. RF participated in the study design, the microbiological sampling in Basel and coordination. LE performed the statistical analysis. JH participated in the study design and coordinated the sampling of the clinical specimen at the University Hospital Basel. AW participated in the study design, coordination and helped to draft the manuscript. JT conceived of the study, and participated in its design and coordination and helped to draft the manuscript. All authors read and approved the final manuscript.

## Funding

This work was supported by the Stiftung Forschung Infektionskrankheiten (SFI Nr 30), Freiwillige Akademische Gesellschaft (FAG), Basel and Margarethe und Walter Lichtenstein-Stiftung, Universität Basel, Basel, Switzerland.

## Pre-publication history

The pre-publication history for this paper can be accessed here:

http://www.biomedcentral.com/1471-2334/12/68/prepub
